# Health and Functional Literacy in Physical Rehabilitation Patients

**DOI:** 10.3928/24748307-20170427-02

**Published:** 2017-06-14

**Authors:** Elizabeth A. Hahn, Susan R. Magasi, Noelle E. Carlozzi, David S. Tulsky, Alex Wong, Sofia F. Garcia, Jin-Shei Lai, Joy Hammel, Ana Miskovic, Sara Jerousek, Arielle Goldsmith, Kristian Nitsch, Allen W. Heinemann

## Abstract

**Background:**

People with disabilities, who face multiple barriers to care, experience health disparities, yet few studies have measured health literacy in this population.

**Objective:**

This study evaluated functional literacy, health literacy, fluid cognitive function, and self-reported health in people who live in community dwellings with spinal cord injury, stroke, or traumatic brain injury.

**Methods:**

Participants with a traumatic spinal cord injury, stroke, or traumatic brain injury, one-year postinjury, and age 18 to 85 years, completed a battery of instruments at three medical centers in the Midwestern U.S.: functional literacy (word recognition, vocabulary knowledge), health literacy (comprehension of prose, document, and quantitative health information), fluid cognitive function (memory, executive function, and processing speed), and patient-reported outcomes (mobility, fatigue, sadness, anxiety, social function, and overall health).

**Key Results:**

There were strong correlations between functional literacy, health literacy, and fluid cognitive function. After adjustment for sociodemographic and clinical characteristics, higher health literacy was associated with better mobility, less anxiety, and better overall health; higher functional literacy was associated with less anxiety and better overall health; and higher fluid cognitive function was associated with better mobility, less sadness, better social function, and better overall health.

**Conclusions:**

To effectively address limited health literacy among people with spinal cord injury, stroke, and traumatic brain injury, and ensure that they are able to be informed partners in their health care, intervention is required at the level of patients, providers, and health care delivery systems. A special consideration is to ensure that health information is both well-targeted to people's health literacy levels and accessible for people with a range of physical, cognitive, and sensory limitations. The multimedia self-administered health literacy measure used in this study could be useful to rehabilitation providers and designers of health information and interfaces. **[*Health Literacy Research and Practice.* 2017;1(2):e71–e85.]**

**Plain Language Summary:**

Health literacy represents people's abilities to obtain, understand, and use health information to make informed decisions about their health and health care. People with disabilities face physical, attitudinal, economic, and structural barriers to care. Consideration of health literacy in rehabilitation practice can enhance the effectiveness of the patient-clinician relationship and help address the needs of this population.

People with disabilities have been called an unrecognized health disparities population (Krahn, Walker, & Correa-De-Araujo, 2015). Although the causes and etiology of disability are diverse, spinal cord injuries, strokes, and traumatic brain injuries accounted for more than one-third of all inpatient rehabilitation cases in 2016 (MedPAC: Medical Payment Advisory Commission, 2016). Although many people make significant improvements during inpatient rehabilitation, many survivors are discharged with long-term physical, functional, and cognitive disabilities. People with disabilities live with a thinner margin of health as they must manage their disabling condition, guard against the onset of secondary conditions, and manage an elevated risk for the development of chronic conditions. People with disabilities experience well-documented physical, attitudinal, and economic barriers to health care and outcomes (Iezzoni, Kurtz, & Rao, 2014). Navigating the health care system requires a high level of sophistication, yet there is a paucity of research about how people with acquired disabilities like spinal cord injury, stroke, and traumatic brain injury obtain, understand, and apply health information (Magasi, Durkin, Wolf, & Deutsch, 2009).

Literacy skills are critical for adults to function effectively in their daily lives (Berkman et al., 2004). The concept of “functional literacy” focuses on the ability to read, write, and speak in English, and to perform quantitative tasks (H.R. 751--102nd Congress, 1991). As evidence accumulated about how low literacy may impair a person's ability to function in the health care environment, and even adversely affect health outcomes, the concept of “health literacy” emerged (Ad Hoc Committee on Health Literacy for the Council on Scientific Affairs & American Medical Association, 1999; Berkman et al., 2004; Parker, Baker, Williams, & Nurss, 1995). Health literacy is “the degree to which individuals can obtain, process, and understand the basic health information and services they need to make appropriate health decisions” (Nielsen-Bohlman, Panzer, & Kindig, 2004). It represents a constellation of skills to perform health-related tasks, including the ability to read and write (print literacy), use quantitative information (numeracy), speak and listen effectively (oral literacy), and obtain information (navigation skills) (Berkman et al., 2011; Nielsen-Bohlman et al., 2004). This definition presents health literacy as a set of individual capacities that tend to be relatively stable over time, although they may improve with educational programs or decline with aging or pathologic processes that impair cognitive function (Baker, 2006; Baker, Gazmararian, Sudano, & Patterson, 2000; Kobayashi, Wardle, Wolf, & von Wagner, 2015). Health literacy may be significantly worse than functional literacy because of the unfamiliar context and vocabulary of the health care system (Ad Hoc Committee on Health Literacy for the Council on Scientific Affairs & American Medical Association, 1999; DeWalt & Pignone, 2005).

Limited health literacy is widespread (Kutner, Greenberg, Jin, & Paulsen, 2006) and is associated with reduced access to health information, poorer health status, limited understanding and use of preventive services, medication errors, increased health care costs and hospitalizations, increased mortality, decreased self-efficacy, and inadequate knowledge and self-care for chronic health conditions (Baker et al., 2002; Berkman et al., 2004; Berkman et al., 2011; DeWalt, Berkman, Sheridan, Lohr, & Pignone, 2004; Macabasco-O'Connell et al., 2011; Nielsen-Bohlman et al., 2004; Peterson et al., 2011; Rudd, Anderson, Oppenheimer, & Nath, 2007). Consideration of health literacy in rehabilitation practice can enhance the effectiveness of the client-provider relationship (Levasseur & Carrier, 2010). However, few studies have measured health literacy in physical rehabilitation populations. One study of patients in the postacute rehabilitation setting and their care partners demonstrated that limited health literacy compromised the ability to understand health quality information and make appropriate decisions about the choice of rehabilitation facilities (Magasi et al., 2009). A single study with individuals with spinal cord injuries indicated that lower health literacy was associated with poorer physical morbidity, but not with mental health morbidity, physical health, or mental health status (Johnston, Diab, Kim, & Kirshblum, 2005).

Some research has documented associations between cognitive abilities and health literacy or functional literacy in selected populations (Baker, Wolf, Feinglass, & Thompson, 2008; Byrd, Sanchez, & Manly, 2005; Chin et al., 2011; Federman, Sano, Wolf, Siu, & Halm, 2009; Kobayashi et al., 2015; Levinthal, Morrow, Tu, Wu, & Murray, 2008; Mõttus et al., 2014; Wolf et al., 2012). No research has examined directly the relationship between health literacy, cognitive abilities, and health outcomes among patients with neurological injuries due to traumatic spinal cord injury, stroke, or traumatic brain injury. Poorer fluid cognitive function (memory, attention, processing speed) (Weintraub et al., 2014) has been reported for all three groups relative to normative standards and controls (Bradbury et al., 2008; Cicerone et al., 2011; Sachdev et al., 2004). Although measurement of cognitive function is part of most standardized rehabilitation programs, assessment of health literacy is not. Given the significant health literacy demands that people with disabilities face while trying to manage multiple medical needs within the complex health care delivery system, greater understanding of the intersection of health literacy, cognitive function, and health outcomes can provide important insights in how to address the needs of this population.

A measure of health literacy is likely to be more closely related to health outcomes than a measure of general (functional) literacy (Baker, 2006). As studies have continued to document associations between literacy and cognitive skills, it may be that cognitive abilities are the causal factor that affects health behaviors and outcomes for people with limited reading ability or limited health literacy (Baker et al., 2000; Barnes, Tager, Satariano, & Yaffe, 2004; Federman et al., 2009; Levinthal et al., 2008; Manly, Schupf, Tang, & Stern, 2005; Mõttus et al., 2014; Wolf et al., 2012). In addition, low health literacy and functional literacy are more prevalent among people with less education (Nielsen-Bohlman et al., 2004). The relationships between health literacy, cognitive ability, and education are complex, and the causal direction of the associations is difficult to tease apart (Morrow et al., 2006; Mõttus et al., 2014; Von Wagner, Steptoe, Wolf, & Wardle, 2009).

The objectives of this study were to (1) describe the functional and health literacy levels of people living in communities with spinal cord injury, stroke, or traumatic brain injury; (2) evaluate associations between functional literacy, health literacy, fluid cognitive function, and education; and (3) estimate the effects of health literacy, functional literacy, and fluid cognitive function on self-reported health. The Behavioral Model for Vulnerable Populations served as the conceptual model for the analyses (Andersen, 1968, 1995; Gelberg, Andersen, & Leake, 2000). This model includes predisposing characteristics (age, gender), enabling resources (functional literacy, health literacy, and fluid cognitive function), and health outcomes (self-reported health).

## Methods

Participants and data for this study were part of a larger project to improve measurement of medical rehabilitation outcomes for persons with spinal cord injury, stroke, or traumatic brain injury (Hammel et al., 2015; Heinemann et al., 2015).

### Participant Recruitment and Enrollment

Participants were recruited at two academic medical centers and one free-standing rehabilitation hospital in the Midwestern part of the United States (the Rehabilitation Institution of Chicago, the University of Michigan in Ann Arbor, and Washington University in St. Louis) after approval from each Institutional Review Board. Target accrual was approximately 200 people in each injury group (spinal cord injury, stroke, and traumatic brain injury). Sites recruited people using research registries, electronic medical records, approved advertisements, within hospital outpatient clinics, and through outreach to patient advocacy organizations.

Eligibility criteria included a traumatic spinal cord injury, stroke, or traumatic brain injury, 1 year postinjury, and age 18 to 85 years. Participants signed an authorization form to release medical information that was used to confirm the participant's injury prior to enrollment. Details about documentation of the injury are available from the authors. After consent, three screening assessments were administered to assess additional eligibility criteria: the ability to see visual stimuli (Snellen score ≥20/100 on the Lighthouse Near Visual Acuity Test [Rosenthal & Lighthouse International, 2015]), the ability to read (the first 10 words on the English Wide Range Achievement Test (WRAT-4) [Wilkinson & Robertson, 2006]), and lack of aphasia (the ability to repeat the main ideas of three stories on the Frenchay Aphasia Screening Test [Enderby, Wood, Wade, & Hewer, 1986]).

### Participant Study Activities

Participants completed a battery of instruments (cognitive function, literacy assessments, and patient-reported outcomes) over a 2-day period. Testing was scheduled in clinical research space at the collaborating institutions, outside of patient care areas. Touchscreen computers (17″ widescreen, 1,440 × 900 resolution) were used with external speakers and/or headphones and additional assistive devices such as a rollerball mouse. Accommodations were made based on individual needs (e.g., assistive devices for writing, interviewer assistance to read questions aloud if people were fatigued or to enter answers into the computer for participants with limited hand function, rest breaks as needed). Participants were free to skip any questions or withdraw from the study at any point. A total honorarium of $80 was provided. In rare instances, if a participant was unable to finish on the second day, she or he came back for a third day and received $20 for travel costs.

### Interviewer Training

To ensure standardized test administration and scoring, all interviewers (3–4 at each site) were trained and certified by either N.E.C. or D.S.T. who are authors of this article. This included at least five practice sessions, and expert observation of one segment of the interviewer's first participant. Each interviewer was recertified 1 year later to ensure that tests continued to be administered in a standardized manner. Interviewers also received training in working with people with disabilities in the context of standardized assessment, including the appropriate provision of reasonable accommodations and assistance. All accommodations were reviewed by an expert in accessibility and measurement (S.R.M.) and a neuropsychologist (N.E.C.).

### Measures and Assessment Procedures

The specific measures that are relevant to this report are summarized here and in **Table A**. Complete study details are reported elsewhere (Heinemann et al., 2015). For each literacy and cognitive test described below, a higher score indicates better performance.

Wide Range Achievement Test, 4th Edition (WRAT-4) (Wilkinson & Robertson, 2006): The WRAT-4 Word Reading Subtest is a list of 55 words and 15 alphabetical letters, ordered by decreasing familiarity and increasing phonological complexity. As the respondent reads the word aloud, the interviewer records whether the word was pronounced correctly or not. The raw score is the total number of correct responses (maximum 70).

Health Literacy Assessment Using Talking Touchscreen Technology (Health LiTT) (Hahn, Choi, Griffith, Yost, & Baker, 2011; Yost et al., 2010): Participants responded to three item types: prose (reading comprehension), document (identify and interpret information presented in charts, graphs or tables), and quantitative (perform arithmetic operations). Each item has a multiple-choice response format, with only one response coded as correct.

Unlike other health literacy tests that require administration by an interviewer, Health LiTT is a novel, self-administered multimedia test that meets psychometric standards for measurement of individual respondents, especially in the low to middle range of health literacy. A 16-item short form was selected for this study. A T-score (mean = 50, standard deviation [SD] = 10) is generated for each participant.

NIH (National Institutes of Health) Toolbox Fluid Cognition Battery (Weintraub et al., 2014): This battery consists of five novel performance-based subtests to assess memory, executive function, and processing speed. An aggregate total score is generated to represent a composite fluid cognition T-score (mean = 100, SD = 15).

NIH Toolbox Oral Reading Recognition Test (Slotkin et al., 2012): This test is a list of words that are shown on a computer. As the respondent reads the word aloud, the interviewer records whether the word was pronounced correctly or not. The test is adaptive; thus, the number of words varies. A variety of adjusted and scaled scores are available; unadjusted scale scores (mean = 100, SD = 15) were used for this study.

NIH Toolbox Picture Vocabulary Test (Slotkin et al., 2012): The respondent is presented with an audio recording of a word and four photographic images on a computer screen, and is asked to select the picture that most closely matches the meaning of the word. The test is adaptive; thus, the number of words varies. A variety of adjusted and scaled scores are available; unadjusted scale scores (mean = 100, SD = 15) were used for this study.

The Rey Auditory Verbal Learning Test (RAVLT) (Schmidt, 1996): A list of words is read aloud by audio recordings on the computer, with assistance from an examiner. The respondent's task is to repeat all the words that she or he can remember, in any order. This procedure is repeated 3 times. The RAVLT is a commonly used measure of a person's ability to encode, consolidate, store, and retrieve verbal information. A variety of adjusted and scaled scores are available; unadjusted scores (mean = 100, SD = 15) were used for this study.

Peabody Picture Vocabulary Test, Fourth Edition (Dunn & Dunn, 2007): The respondent is presented with four colored illustrations for each of 10 items. She or he selects the picture that best represents the meaning of a stimulus word that is presented orally by the interviewer. Scores are based on the number of correct responses (maximum 228).

Sociodemographic information and self-reported physical, mental, social, and overall health were obtained from other instruments completed by participants, including Participation Survey/Mobility (PARTS/M) (Gray, Hollingsworth, Stark, & Morgan, 2006), Neuro-QoL (Quality of Life in Neurological Disorders) Mobility (Cella et al., 2011), PROMIS (Patient-Reported Outcomes Measurement Information System) Fatigue (Schneider, Choi, Junghaenel, Schwartz, & Stone, 2013), NIH Toolbox Sadness and Fear Affect (Salsman et al., 2013), and PROMIS Social Function (Ability to Participate in Social Roles and Activities) (Hahn et al., 2014). For each of the self-reported health measures, a higher score represents better health for positive concepts (mobility, social function) or poorer health for negative concepts (fatigue, sadness, fear affect).

## Statistical Analysis

Characteristics and measures of literacy, cognition, and self-reported health were compared between the three groups of participants (spinal cord injury, stroke, traumatic brain injury) using analysis of variance, a chi-square test or a Fisher's exact test, and effect sizes (Ferguson, 2009). The Tukey-Kramer test was used to adjust for posthoc pairwise comparisons. The spinal cord injury group was expected to have better fluid cognition and health literacy, and poorer mobility, than the stroke and traumatic brain injury groups. No group differences were hypothesized for mental and social health. Because overall health incorporates physical, mental, and social health, no hypotheses were made. Associations between education, literacy, and cognition were evaluated with Pearson or Spearman correlations. Mean Health LiTT scores were compared using analysis of variance across categories of self-reported health status within injury group.

Multivariable linear (Kleinbaum, Kupper, & Muller, 1988) or multinominal logit regression (Agresti, 2002) was used to explore the effects of health literacy, functional literacy, and fluid cognitive function on self-reported health. One measure of functional literacy and one measure of fluid cognitive function was chosen by the investigators for these analyses. Separate sets of analyses were conducted for each of the following dependent variables: mobility, fatigue, sadness, fear affect (anxiety), ability to participate in social roles and activities, and overall health (poor/fair vs. good vs. very good/excellent). All models included sociodemographic and clinical characteristics (injury group, gender, age, ethnicity/race, and benefits [none vs. any]). Because of strong correlations between functional literacy, health literacy, and fluid cognitive function, only one of these covariates was added to each model. A sample size of 600 provided 80% power to detect an *r*-squared as small as 0.02 with six covariates. A nominal significance level of 0.05 was used. Analyses were conducted with SPSS version 20 software.

## Results

All participants were enrolled at least 1 year postinjury; mean time since injury was 12 years for the spinal cord injury group, 3 years for stroke, and 6 years for traumatic brain injury. About half of the participants in each group had a severe injury; specifically, 49% of the spinal cord injury participants had complete paraplegia or tetraplegia, 44% of the stroke participants had a severe stroke, and 54% of the traumatic brain injury participants had a severe diagnosis.

Participants in the spinal cord injury and traumatic brain injury groups were predominantly non-Hispanic White men, with mean ages of 46 and 40 years, respectively (**Table 1**). The stroke group was slightly older, with equal numbers of women and men, and 48% non-Hispanic Blacks. About one-third in each group had a high school or lower education, and about one-third were currently married or in a committed partner relationship.

Descriptive information for health literacy, functional literacy, fluid cognitive function, and self-reported health is summarized in **Table 2**. The three injury groups differed in most of these measures. The stroke group had the lowest levels of health literacy, functional literacy, and fluid cognitive function, and the poorest overall health.

Correlations between Health LiTT and the four functional literacy measures ranged from 0.57 to 0.65 (see fourth column of **Table 3**). Correlations among the functional literacy measures were slightly higher (0.60 to 0.86). Correlations between the health/functional literacy measures and the two fluid cognitive function measures were much lower (0.26 to 0.49; see bottom two rows of **Table 3**). Correlations between literacy and education were 0.40 to 0.48 (see third column of **Table 3**). These patterns were similar within each of the three injury groups (spinal cord injury, stroke, traumatic brain injury).

After adjusting for injury group, gender, age, ethnicity/race, and benefits, higher health literacy (Health LiTT) was significantly associated with better mobility, less fear affect (anxiety), and better overall health; higher functional literacy (WRAT) was significantly associated with less fear affect (anxiety) and better overall health; and higher fluid cognitive function was significantly associated with better mobility, less sadness, better ability to participate in social roles and activities, and better overall health (**Table 4**). There were no associations between health literacy, functional literacy, or fluid cognitive function and fatigue.

**Figure 1.**
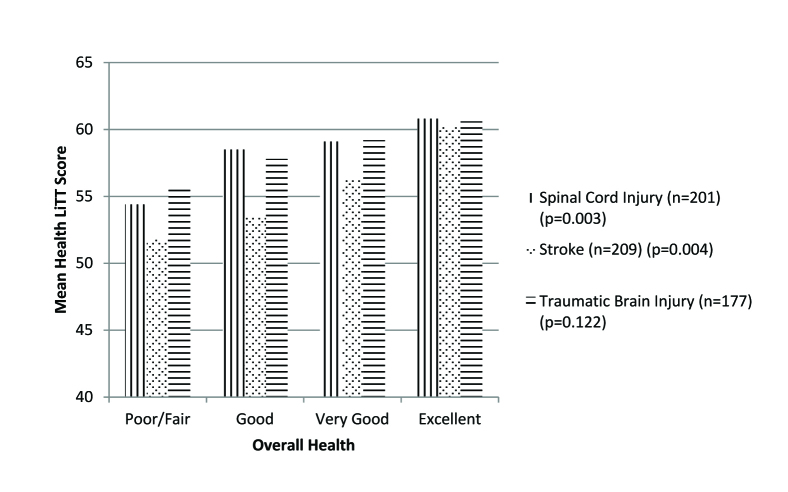
Mean Health LiTT scores by health status and injury group. Health LiTT = Health Literacy Assessment Using Talking Touchscreen Technology.

Mean Health LiTT scores increased across levels of self-reported health, within each injury group (spinal cord injury, F(3,197) = 4.69, *p* = .003; stroke, F (3,205) = 4.63, *p* = .004; traumatic brain injury, F (3,173) = 1.96, *p* = .122; **Figure 1**).

## Discussion

This was the first study to evaluate health literacy, functional literacy, fluid cognitive function, and self-reported health in physical rehabilitation populations. Participants in three injury groups (spinal cord injury, stroke, and traumatic brain injury) differed in most sociodemographic characteristics, health literacy, most functional literacy measures, both fluid cognitive function measures, and some self-reported health outcomes. The stroke group had the lowest levels of health literacy, functional literacy, and fluid cognitive function, and the poorest overall health. After adjusting for injury group, gender, age, ethnicity/race, and current benefits, higher health literacy, functional literacy, and fluid cognitive function each was significantly associated with better overall health and with one or more measures of physical, mental, or social health. Specifically, higher health literacy was associated with better mobility and less anxiety, higher functional literacy was associated with less anxiety, and higher fluid cognitive function was associated with better mobility, less sadness, and better ability to participate in social roles and activities. There were no associations with fatigue. At increasing levels of self-reported overall health, participants had higher average health literacy than those in the next lower level, similar to national findings (Kutner et al., 2006). Health literacy was strongly correlated with fluid cognitive function (range, r = 0.463 to 0.494). Similar findings were reported in a large study of older adults (age 55 to 74 years) (Wolf et al., 2012), and in a diverse sample of primary care patients (Yost, DeWalt, Lindquist, & Hahn, 2013).

There are some limitations to this study. These three samples are not diverse enough to generalize to the US population of people living with spinal cord injury, stroke, or traumatic brain injury. By selection, participants were living in the community and were at least 1 year postinjury and therefore may not reflect the experiences of people in more acute rehabilitation. This limitation is significant since the most substantial gains postinjury occur within the first 12 months for these groups. Future research may be needed to establish reliability and validity of the Health LiTT measurement system among inpatient rehabilitation. Additionally, the exclusion of people with aphasia may have limited people with left hemispheric strokes and thus limited the representativeness of the stroke sample. The demands of the 2-day testing session may have led to self-selection bias towards people with higher levels of health literacy and cognitive function. There were no measures of access to, or satisfaction with, rehabilitation decision-making. Because this was a cross-sectional study, the results cannot inform understanding of any casual associations between health literacy, fluid cognitive ability, and patient-reported outcomes.

Health literacy is important because it represents people's abilities to obtain, understand, and use health information to make informed decisions about their health and health care (Baker, 2006; Nielsen-Bohlman et al., 2004). People with disabilities are increasingly recognized as an important health disparities population who face multiple physical, attitudinal, economic, and structural barriers to care (Krahn et al., 2015). To effectively address limited health literacy among people with spinal cord injury, stroke, and traumatic brain injury, and ensure that they are able to be informed partners in their health care, intervention is required at the level of individual patients, providers, and health care delivery systems (Magasi et al., 2015). For example, rehabilitation providers can help people regain and acquire the skills, knowledge, and ability to understand and use health information. Health care providers can ensure that information is presented in ways that people with disabilities can use and understand; this is true both for individual practitioners and health care systems as they prepare and present information in a variety of in-person, online, and print formats. A special consideration when addressing the health information needs of people with disabilities is to ensure that health information is both well-targeted to people's health literacy levels and accessible for people with a range of physical, cognitive, and sensory limitations.

Health literacy is rarely addressed in rehabilitation research or clinical practice. The health literacy measure used in this study could be useful to rehabilitation providers and designers of health information and interfaces. The Health LiTT measurement system uses multimedia health information technology, meets high psychometric standards for measuring health literacy in individual respondents (Hahn et al., 2011), and is publicly available (www.healthlitt.org). It could be a valuable tool for identifying patients in need of interventions to address low health literacy. Additionally, self-administration enables efficient measurement of health literacy while placing limited administration burden on clinicians and helping to avoid the potential stigma patients may feel related to low literacy. Health LiTT also provides a measurement strategy to estimate the size of the population at risk from low health literacy at the clinic or health system level to inform how health information is developed and shared with patients. For example, customizable design elements could be built into health information to enable different groups of potential users to access, understand, and effectively use the information in decision-making. Finally, the Health LiTT measurement system provides reliable and valid scores that can be used in testing interventions to help build an evidence-informed approach to address diverse health literacy skills among people with spinal cord injury, stroke, or traumatic brain injury. Better integration of health literacy, health equity, and patient-centered care initiatives (Hasnain-Wynia & Wolf, 2010; Paasche-Orlow & Wolf, 2010) would help to shift the focus from the negative effects of low health literacy to a positive model of how health literacy can be used to improve health (Pleasant, Cabe, Patel, Cosenza, & Carmona, 2015).

## Figures and Tables

**Table 1:** Sociodemographic Characteristics of Study Participants by Injury Type

**Sociodemographic Characteristic**	**Injury Type**			***p* Value^a^**	**Test Statistic**	**Effect Size^b^**

	**Spinal Cord Injury**	**Stroke**	**Traumatic Brain Injury**			

	(*n* = 209)	(*n* = 211)	(*n* = 184)			

Female gender	45 (22%)	106 (50%)	66 (36%)	< .001	*X*^2^(2,*n* = 604) = 37.58	0.25

Age in years, mean (SD)	46 (14)	56 (13)	40 (17)	< .001	F(2,601) = 65.94	0.17

*Ethnicity, race*						

Hispanic (any race)	18 (9%)	11 (5%)	13 (7%)	< .001	*X*^2^(6,*n* = 600) = 53.85	0.21
Non-Hispanic, Black	60 (29%)	100 (48%)	29 (16%)			
Non-Hispanic, White	125(60%)	86 (41%)	128 (70%)			
Non-Hispanic, Other	5 (2%)	12 (6%)	13 (7%)			

*Highest education*						

Less than HS	18 (9%)	24 (11%)	20 (11%)	.961	*X*^2^(6,*n* = 604) = 1.48	0.04
HS/GED	48 (23%)	46 (22%)	42 (23%)			
Some college	76 (36%)	80 (38%)	65 (35%)			
College degree	67 (32%)	61 (29%)	57 (31%)			

Married/partner	68 (36%)	69 (35%)	54 (33%)	.884	*X*^2^(2,*n* = 552) = 0.25	0.02

*Current benefits received*						

SSI, SDI, or SS	36 (19%)	55 (26%)	23 (14%)	<.001	Table probability <0.001	0.26
Medicare	47 (24%)	51 (24%)	27 (16%)			
Medicaid	74 (38%)	52 (25%)	30 (18%)			
Independent living center services	10 (5%)	1 (1%)	3 (2%)			
None of the above	28 (14%)	49 (24%)	82 (50%)			

Note. Entries in the table represent the number of participants (percentage), unless otherwise specified. The amount of missing data varied, and was excluded from the table. Spinal Cord Injury group: *n* = 1 missing ethnicity/race, *n* = 18 missing marital/partner status, *n* = 14 missing current benefits. Stroke group: *n* = 2 missing ethnicity, *n* = 13 missing marital/partner status, *n* = 3 missing current benefits. Traumatic Brain Injury group: *n* = 1 missing ethnicity/race, *n* = 21 missing marital/partner status, *n* = 19 missing current benefits. GED = General Educational Development Test (High School Equivalency Diploma); HS = high school; SD = standard deviation, SDI = state disability insurance; SS = social security, SSI = supplemental security income.

a *p* value: unadjusted analysis of variance, chi-square test, or Fisher's exact test.

b Effect size: Cramer's V for all characteristics except age, which used omega-squared.

**Table 2:** Literacy, Cognition, and Self-Reported Health by Injury Type

**Literacy, Cognition, and Self-Reported Health**	**Injury Type**			***P* Value^a^**	**Test Statistic**	**Effect Size^b^**

	**Spinal Cord Injury**	**Stroke**	**Traumatic Brain Injury**			
	(*n* = 209)	(*n* = 211)	(*n* = 184)			

**Instrument/measure^c^**						
*Health literacy*						

Health LiTT	58.1 (7.1)^d^	53.6 (9.2)	57.8 (7.5)^d^	<.001	F(2,601) = 20.24	0.06

*Functional literacy*						

WRAT-4 Word Reading Subtest	56.8 (8.6)^d,e^	55.2 (9.7)^e^	57.7 (7.1)^d^	.018	F(2,593) = 4.07	0.01

NIH Toolbox Oral Reading Recognition Test	103.0 (9.4)	100.9 (9.5)	103.8 (8.3)	.006	F(2,582) = 5.18	0.01

NIH Toolbox Picture	103.5 (12.5)	101.4 (14.0)	102.5 (10.4)	.214	F(2,584) = 1.54	0.001

Vocabulary Test PPVT-4	203.6 (18.9)^d^	198.1 (25.1)^e^	202.6 (16.1)^d,e^	.017	F(2,590) = 4.12	0.01

*Fluid cognitive function*						

NIH Toolbox Fluid Cognition Composite Score	99.4 (10.6)^d^	86.6 (14.2)	97.9 (14.1)^d^	<.001	F(2,497) = 48.51	0.16

RAVLT	99.4 (15.9)^d^	92.7 (14.8)	98.9 (17.1)^d^	<.001	F(2,556) = 10.80	0.03

**Self-reported health**						
*Physical health*						

Neuro-QoL Mobility	30.7 (9.0)	45.7 (10.2)	51.1 (10.3)	<.001	F(2,542) = 204.52	0.43

PROMIS Fatigue	49.2 (9.0)	49.1 (9.3)	50.1 (10.2)	.608	F(2,568) = 0.50	−0.002

*Mental health*						

NIH Toolbox Sadness	104.5 (16.0)	104.1 (16.3)	105.1 (17.6)	0.829	F(2,572) = 0.19	−0.002

NIH Toolbox Fear Affect (Anxiety)	102.7 (14.8)	103.2 (17.2)	104.2 (18.2)	0.694	F(2,572) = 0.36	−0.002

*Social health*						

PROMIS Ability to Participate in Social Roles and Activities	48.9 (7.5)	49.1 (9.3)^d^	52.9 (9.1)^d^	<0.001	F(2,562) = 10.50	0.03

*Overall health*						

Poor	1 (1%)	13 (6%)	7 (4%)	<0.001	X^2^(8,n = 587) = 44.90	0.20
Fair	33 (16%)	66 (31%)	26 (15%)			
Good	84 (42%)	85 (41%)	77 (43%)			
Very Good	62 (31%)	31 (15%)	58 (33%)			
Excellent	21 (10%)	14 (7%)	9 (5%)			

Note. Entries in the table represent the mean (and standard deviation) or number of participants (percentage). The amount of missing data varied and was excluded from the table. Spinal Cord Injury group: *n* = 3 missing WRAT-4, *n* = 5 missing NIH Toolbox Oral Reading test, *n* = 4 missing NIH Toolbox Picture Vocabulary test, *n* = 5 missing PPVT-4, *n* = 52 missing NIH Toolbox Fluid Cognition composite score, *n* = 14 missing RAVLT, *n* = 5 missing Overall Health, *n* = 25 missing Neuro-QoL Mobility, *n* = 13 missing PROMIS Fatigue, *n* = 13 missing NIH Toolbox Sadness, *n* = 13 missing NIH Toolbox Fear Affect, *n* = 17 missing PROMIS Ability to Participate in Social Roles and Activities. Stroke group: *n* = 3 missing WRAT-4 test, *n* = 8 missing NIH Toolbox Oral Reading Recognition test, *n* = 6 missing NIH Toolbox Picture Vocabulary test, *n* = 2 missing PPVT-4, *n* = 32 missing NIH Toolbox Fluid Cognition composite score, *n* = 15 missing RAVLT, *n* = 34 missing Overall Health, *n* = 11 missing Neuro-QoL Mobility, *n* = 9 missing PROMIS Fatigue, *n* = 7 missing NIH Toolbox Sadness, *n* = 7 missing NIH Toolbox Fear Affect, *n* = 8 missing PROMIS Ability to Participate in Social Roles and Activities. Traumatic Brain Injury group: *n* = 2 missing WRAT-4 test, *n* = 6 missing NIH Toolbox Oral Reading Recognition test, *n* = 7 missing NIH Toolbox Picture Vocabulary test, *n* = 4 missing PPVT-4, *n* = 20 missing NIH Toolbox Fluid Cognition composite score, *n* = 16 missing RAVLT, *n* = 7 missing Overall Health, *n* = 23 missing Neuro-QoL Mobility, *n* = 11 missing PROMIS Fatigue, *n* = 9 missing NIH Toolbox Sadness, *n* = 9 missing NIH Toolbox Fear Affect, *n* = 14 missing PROMIS Ability to Participate in Social Roles and Activities. Neuro-QoL = Quality of Life in Neurological Disorders; NIH = National Institutes of Health; PPVT-4 = Peabody Picture Vocabulary Test; PROMIS = Patient-Reported Outcomes Measurement Information System; RAVLT = Rey Auditory Verbal Learning Test; WRAT-4 = English Wide Range Achievement Test.

a p Value: unadjusted analysis of variance or chi-square test.

b Effect size: Omega-squared for all measures except overall health, which utilized Cramer's V.

c See text and **Table A** for a description of each instrument/measure.

d,e Mean values with the same superscript were not significantly different from one another (Tukey-Kramer test).

**Table 3:** Correlations Among Education, Health Literacy, Functional Literacy, and Fluid Cognitive Function Measures

		**Education**	**Health Literacy**	**Functional Literacy**				**Fluid Cognitive Function**
			**Health LiTT**	**Wide Range Achievement Test-4 Word Reading Subtest**	**NIH Toolbox Oral Reading Recognition Test**	**NIH Toolbox Picture Vocabulary Test**	**Peabody Picture Vocabulary Test**	**NIH Toolbox Fluid Cognition Battery**
**Health Literacy**	**Health LiTT**	0.40 (*n* = 604)						
**Functional Literacy**	**Wide Range Achievement Test-4 Word Reading Subtest**	0.45 (*n* = 596)	0.58 (*n*=596)					
	**NIH Toolbox Oral Reading Recognition Test**	0.48 (*n* = 585)	0.62 (*n* = 585)	0.86 (*n* = 581)				
	**NIH Toolbox Picture Vocabulary Test**	0.48 (*n* = 587)	0.65 (*n* = 587)	0.65 (*n* = 580)	0.72 (*n* = 582)			
	**Peabody Picture Vocabulary Test-4**	0.46 (*n* = 593)	0.57 (*n* = 593)	0.60 (*n* = 590)	0.60 (*n* = 578)	0.82 (*n* = 578)		
**Fluid Cognitive Function**	**NIH Toolbox Fluid Cognition Battery**	0.14 (*n* = 500)	0.49 (*n* = 500)	0.28 (*n* = 499)	0.31 (*n* = 498)	0.28 (*n* = 497)	0.26 (*n* = 496)	
	**Rey Auditory Verbal Learning Test**	0.18 (*n* = 559)	0.46 (*n* = 559)	0.30 (*n* = 557)	0.33 (*n* = 548)	0.33 (*n* = 548)	0.29 (*n* = 558)	0.45 (*n* = 473)

Note. Spearman correlation coefficients were estimated for analyses with education categories; Pearson correlation coefficients were estimated for all other analyses. All correlations were significantly different from 0 at *p* < .05 (two-tailed). Health LiTT = Health Literacy Assessment Using Talking Touchscreen Technology; NIH = National Institutes of Health.

**Table 4:** Multivariable Regression Results

Endpoint (dependent variable)						
*Linear regression*						
	**Health LiTT Adjusted Coefficient**	**Model F-Statistic**	**WRAT Adjusted Coefficient**	**Model F-Statistic**	**Fluid Cognitive Function Adjusted Coefficient**	**Model F-Statistic**
*Physical health*						
Neuro-QoL mobility	0.169^a^	56.7^a^				
			0.058	55^a^		
					0.266^a^	51.2^a^
PROMIS fatigue	0.037	2^b^				
			0.034	1.9		
					−0.015	1.4
*Mental health*						
NIH toolbox sadness	−0.139	1.6				
			−0.146	1.6		
					−0.159^b^	1.5
NIH toolbox fear affect	−0.265^b^	2.8^a^				
			−0.279^a^	3^a^		
					−0.88	2.1^b^
*Social health*						
PROMIS ability to participate in social roles and activities	0.069	3.8^a^				
			0.049	3.7^a^		
					0.101^a^	4.4^a^
*Multinomial logit regression*						
	**Health LiTT Adjusted Coefficient**	**Likelihood Ratio Chi-Square**	**WRAT Adjusted Coefficient**	**Likelihood Ratio Chi-Square**	**Fluid Cognitive Function Adjusted Coefficient**	**Likelihood Ratio Chi-Square**
Overall health		79.1^b^		78.4^a^		54.4^a^
Very good/excellent vs. poor/fair	1.061^a^					
Good vs. poor/fair	1.040^a^					
Very good/excellent vs. poor/fair			1.053^a^			
Good vs. poor/fair			1.031^b^			
Very good/excellent vs. poor/fair					1.031^b^	
Good vs. poor/fair					1.017	

Note. Entries in the table represent adjusted regression coefficients (or adjusted odds ratios) and model test statistics for each endpoint. All models included injury group, gender, age, ethnicity/race, and benefits. See text for details. Health LiTT = Health Literacy Assessment Using Talking Touchscreen Technology; NIH = National Institutes of Health; PROMIS = Patient-Reported Outcomes Measurement Information System; QoL = quality of life; WRAT = Wide Range Achievement Test.

a *p* < .01.

b *p* < .05.

**Table A:** Literacy and Cognitive Function Measures (in Order of Administration)

**Construct**	**Instrument^a^**	**Measurement Task (Number of Items)**	**Mode**	**Method**	**Approximate Time for Completion (Minutes)**
Functional literacy	Wide Range Achievement Test-4 Word Reading Subtest	Word recognition (55 words and 15 letters)	Interviewer-guided performance	Paper teleform	5
Health literacy	Health Literacy Assessment Using Talking Touchscreen Technology	Comprehension of prose, document, and quantitative health information (16 items)	Self-administered	Talking touchscreen	10
Fluid cognitive function	NIH Toolbox Fluid Cognition Battery	Flanker Inhibitory Control and Attention Test (varies), List Sorting Working Memory (varies), Dimensional Change Card Sort Test (varies), Pattern Comparison Processing Speed (varies), Picture Sequence Memory Test (varies)	Interviewer-guided performance	Computer	60
Functional literacy	NIH Toolbox Oral Reading Recognition Test	Word recognition (computer-adaptive test)	Interviewer-guided performance	Computer	
Functional literacy	NIH Toolbox Picture Vocabulary Test	Vocabulary knowledge (computer-adaptive test)	Self-administered	Computer	
Fluid cognitive function	Rey Auditory Verbal Learning Test	Short-term verbal memory	Interviewer-guided performance	Computer	7
Functional literacy	Peabody Picture Vocabulary Test-4	Vocabulary knowledge (10 items)	Interviewer-guided performance	Paper teleform	10

a See text for description of each instrument. NIH = National Institutes of Health.
